# Supplementary figures and images for: Systematics, distribution patterns and historical biogeography of the Central America wandering spider genus Kiekie Polotow & Brescovit, 2018 (Araneae: Ctenidae)
Source: PeerJ. 2024 Apr 29;12:e17242. doi: 10.7717/peerj.17242 (PMC11064872; doi:10.7717/peerj.17242)

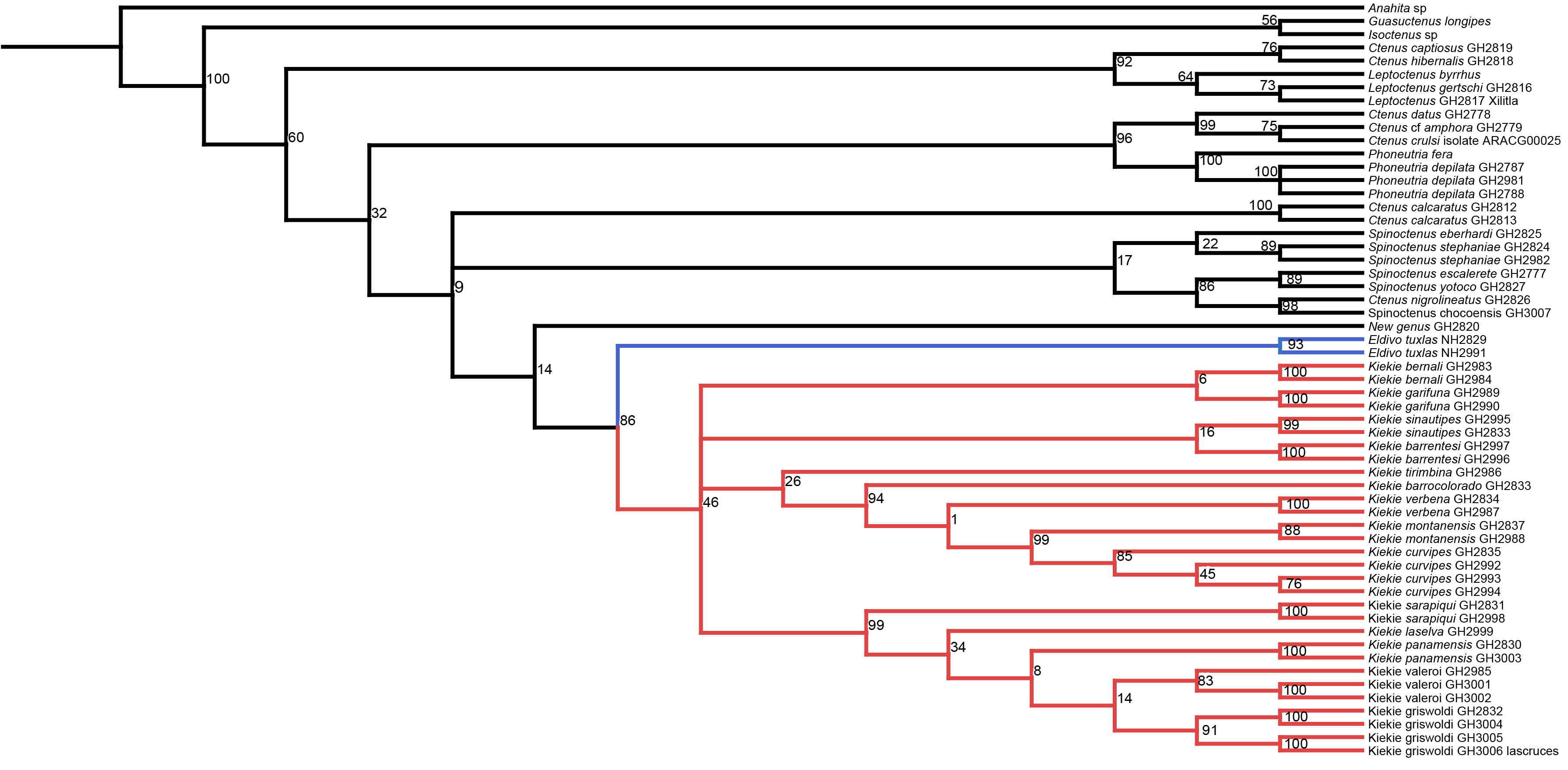

Supplement: Supplemental Information 3 — Nodes with jackknife values resampling expressed as GC ‘‘Group present/Contradicted’’ frequency differences. Blue clade: Eldivo, red clade: Kiekie. [file peerj-12-17242-s003.png]

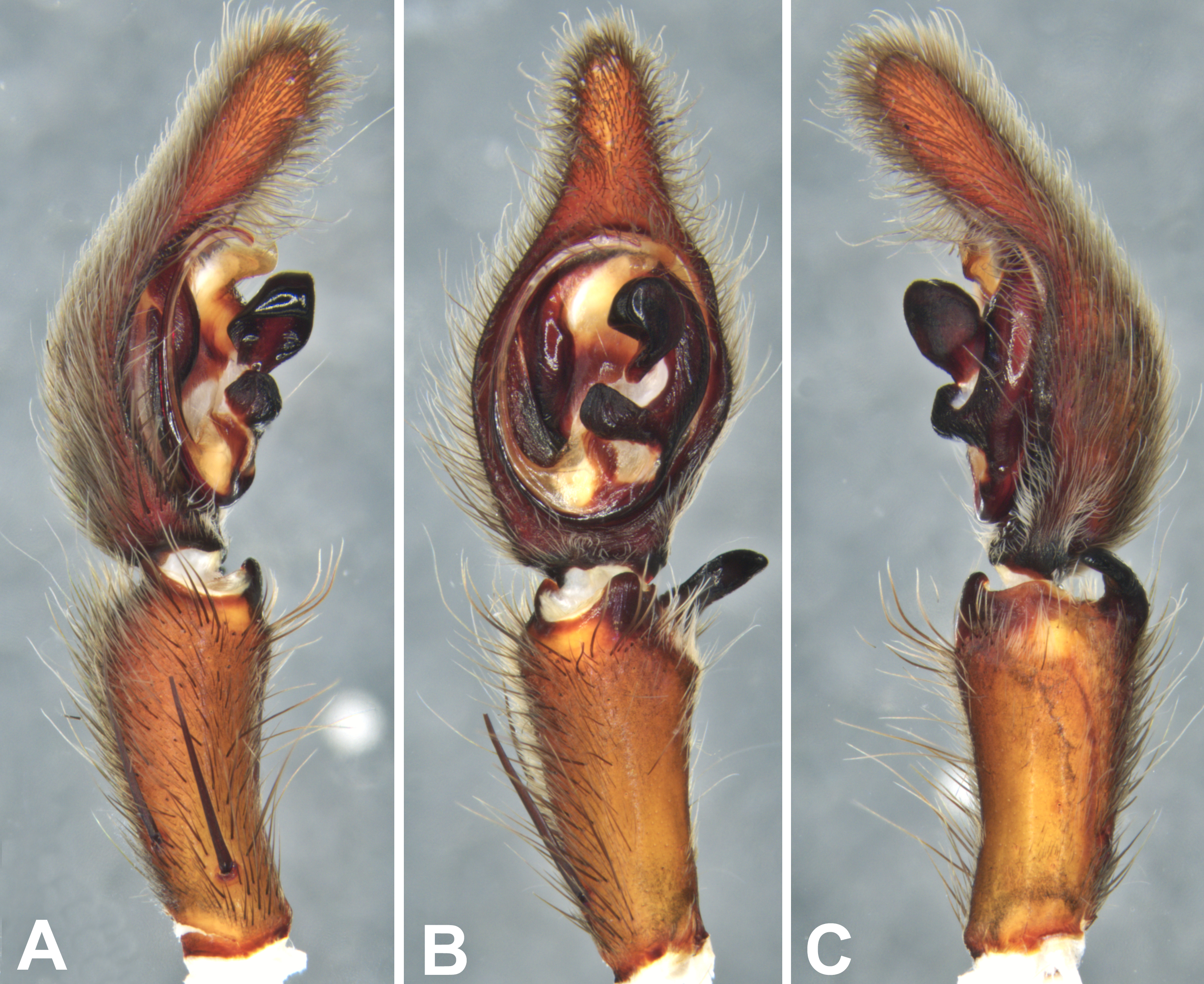

Supplement: Supplemental Information 4 — A, ventral view; B, prolateral view; C, retrolateral view; scale bar = 1.00 mm. [file peerj-12-17242-s004.png]
